# National trends in burden of enteric infections in China: Shifts from 1990 to 2021

**DOI:** 10.1016/j.imj.2025.100213

**Published:** 2025-10-13

**Authors:** Huichao Wu, Menglong Li, Zuolin Lu, Jing Huang, Fuqiang Cui, Ruitai Shao

**Affiliations:** a Department of Hospital Infection Control, Peking Union Medical College Hospital, Chinese Academy of Medical Sciences and Peking Union Medical College, Beijing 100730, China; b School of Population Medicine and Public Health, Chinese Academy of Medical Sciences and Peking Union Medical College, Beijing 100005, China; c Evidence-Based Medicine Center, Beijing Luhe Hospital, Capital Medical University, Beijing 101149, China; d Center for Infectious Diseases and Policy Research and Global Health and Infectious Diseases Group, Peking University, Beijing 100191, China

**Keywords:** Enteric infection, China, Trend, Diarrheal disease, Typhoid and paratyphoid fever

## Abstract

**Background:**

Enteric infections impose a substantial global health burden annually, especially in countries with inadequate sanitation. This study aims to assess the trends in the burden of enteric infections in China.

**Methods:**

Prevalence, incidence, deaths, and disability-adjusted life years (DALYs) attributed to enteric infections by etiology in China were leveraged from the Global Burden of Disease Study 2021. Temporal trends in the burden of enteric infections from 1990 to 2021 were determined by percent changes and average annual percent change (AAPC). Decomposition analysis for percent changes was conducted to understand the contributions of demographic and epidemiological changes.

**Results:**

In 2021, China bore 75.15 million incidence cases and 5,590 deaths from enteric infections, with age-standardized incidence and mortality rates of 59.04 per 100,000 and 0.44 per 100,000, respectively. Substantial decreases, mainly driven by epidemiological changes, were observed in incidence cases (−74.12%), deaths (−94.09%), and DALYs (−95.60%) from 1990 to 2021. Simultaneously, age-standardized incidence, mortality, and DALY rates showed decreasing trends for enteric infections and the subtypes in China. Diarrheal diseases were consistently the main constituent of the burden of enteric infections in China.

**Conclusions:**

From 1990 to 2021, China has made significant strides in the control of enteric infections. This study underscores the need for continued efforts for the improvement of the health system, and enhanced interventions and health promotion strategies for both young and older populations.

## Highlights

• China had 75.15 million new cases and 5590 deaths from enteric infections in 2021
• Substantial decreases showed in number and rates of enteric infections, 1990–2021
• Diarrheal diseases were the main contributor of enteric infections burden in China
• A dual age pattern was identified from higher incidence rates and DALY rates

## Graphical abstract

**Figure:**
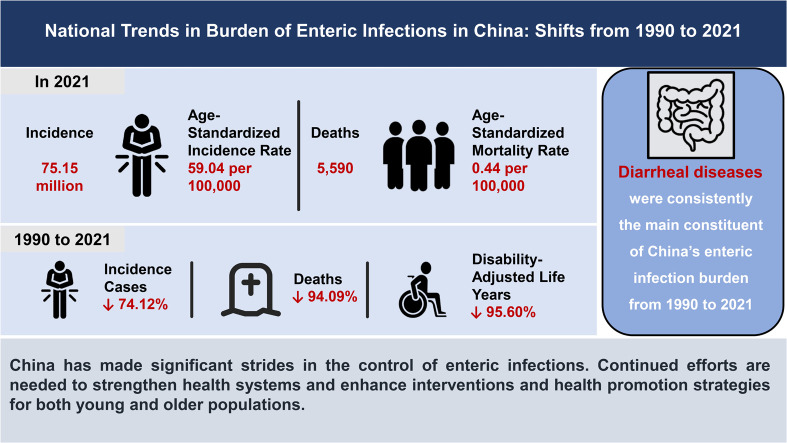

## 1 Introduction

Enteric infections, encompassing diarrheal diseases, typhoid and paratyphoid fever, invasive non-typhoidal *Salmonella* (iNTS) infections, and others, are caused by viruses, bacteria, parasites, or chemical substances and typically transmitted via contaminated food or water.^1^^,^^2^ Enteric infections affect millions of population annually and impose a substantial global health burden, especially in low- and middle-income countries with developing economies and inadequate sanitation.^2^, ^3^, ^4^ Unsafe water, sanitation, and handwashing practices contribute to the disease burden, underscoring the role of healthcare infrastructure in mitigating the impact of enteric infections.^2^^,^^4^^,^^5^ Studies further revealed that enteric pathogens such as norovirus, rotavirus, *Salmonella, Campylobacter, Escherichia coli,* and *Cryptosporidium* are common causes leading to hospitalization in children and the elderly, highlighting the importance of healthcare management in addressing these infections.^2^^,^^6^ Foodborne and waterborne enteric infections are preventable, and the development of health systems and the improvement of public health policies can significantly impact the management and prevention of enteric infections.^3^^,^^7^

China, with its large population and diverse geographical conditions, has experienced a range of challenges in managing the burden of enteric infections.^8^^,^^9^ With the socio-economic, urbanization and lifestyle transitions, which can impact the transmission and control of enteric infections, the epidemiological trends and disease spectrum of enteric infections are also changing in China.^7^, ^8^, ^9^ In recent years, the Chinese government has increased investment in public health and health system improvement, particularly in food safety regulation, drinking water hygiene improvement, and infectious disease control, leading to a decreasing trend in the incidence of enteric infections.^8^^,^^10^ Understanding the burden of enteric infections and dynamically tracking the changing trends of enteric infections and the epidemiological characteristics of different etiologies of enteric infections are essential for the improvement of effective prevention and control strategies.

This study leveraged the Global Burden of Disease Study (GBD) 2021 database (https://vizhub.healthdata.org/gbd-results/) to assess the prevalence, incidence, deaths, and disability-adjusted life years (DALYs) of enteric infections in China from 1990 to 2021. By tracking the burden, temporal trends and drivers of enteric infections at the nation scale, we aimed to provide multifaceted implications of the continuing efforts in management of enteric infections and to offer data-driven insights for the adjustment of prevention and control measures.

## 2 Materials and methods

### 2.1 Data source

The GBD 2021 provides comprehensive estimates on the prevalence, incidence, mortality and DALYs for various diseases in 204 countries and territories and 811 subnational locations.^11^^,^^12^ The GBD 2021 represents the most comprehensive and methodologically standardized observational epidemiological study to date. The data used in our study were searched by measure, metric, cause, and age in China, globally, in the 21 regions, and in the four health system levels from 1990 to 2021. This study used de-identified public-accessible data from the GBD 2021 study. Thus, the ethical approval was waived by the institutional review board.

### 2.2 Disease definition

This study specifically focused on the burden of enteric infections, which aggregate cause incorporates deaths and disabilities resulting from infections to the intestines caused by a variety of different etiologies.^1^ The International Classification of Diseases, 10th Revision (https://icd.who.int/browse10/2019/en) codes used for diarrheal diseases included A00–A00.9, A02–A02.0, A02.8–A07, A07.2–A07.4, A08–A08.8, A09, and K52.1. The other specific etiologies included typhoid and paratyphoid fever (A01–A01.4), iNTS (A02.1–A02.29), and other enteric infectious diseases (A07.0–A07.1, A07.8–A07.9, A80–A80.9, and B91).^2^^,^^3^^,^^11^

### 2.3 Disease burden

The GBD database provided the estimation of disease burden measures using the Bayesian meta-regression method, known as DisMod-MR 2.1, and recognized for its reliability and consistency.^11^ The incidence, prevalence, mortality, and DALY rate are calculated by dividing the number of new cases, the total number of cases, the estimated number of deaths, and the number of DALYs by the corresponding population, respectively. All rates by a 5-year interval are presented per 100,000 population.^2^^,^^3^^,^^11^

### 2.4 Statistical analysis

In this study, the crude rates were used to quantify the real-world burden, while the age-standardized rates (ASRs) were used to conduct valid comparisons across time by accounting for demographic shifts. ASR was determined by the direct method to the GBD standard population and calculated by aggregating the products of age-specific rates ($a_{i}$) and corresponding population counts ($W_{i}$) within each 5-year interval, and the dividing by the sum of standard population weights. The formula (1) is expressed as follows:

(1) $$ASR=\frac{\sum_{i=1}^{A}a_{i}W_{i}}{\sum_{i=1}^{A}W_{i}}\times 100,000$$

The uncertainty intervals (UIs) were generated from 1,000 iterations in both the ensemble and meta-regression models, resulting in 95% UIs defined by the 2.5th and 97.5th percentiles of the 1,000 estimated draws provided by the GBD database.

The percent changes in prevalence cases, incidence cases, deaths, and DALYs between the years 1990 and 2021 were calculated using the following formula (2):

(2) $$\mathrm{Percent}\;\mathrm{changes}=\frac{{\mathrm{Values}}_{2021}-{\mathrm{Values}}_{1990}}{{\mathrm{Values}}_{1990}}\times 100\%$$

Average annual percent change (AAPC) was used to assess temporal trends in age-standardized prevalence, incidence, mortality, and DALY rates (age-standardized prevalence rate [ASPR], age-standardized incidence rate [ASIR], age-standardized mortality rate [ASMR], and age-standardized DALY rate [ASDR]) of enteric infections from 1990 to 2021. AAPC with the 95% confidence interval (CI) was derived from log-linear Joinpoint models that best fit the data.^12^, ^13^, ^14^ The AAPC is determined using the formula (3), where $\beta_{i}$ denotes the slope coefficient for the $i^{th}$ segment, i denotes the segments within the specified range of years, and $w_{i}$ denotes the length of each segment within the year range:

(3) $$\text{AAPC}=[\exp\left(\frac{\sum w_{i}\beta_{i}}{\sum w_{i}}\right)-1]\times 100$$

Additionally, a decomposition analysis was conducted to determine the drivers of changes in prevalence cases, incidence cases, deaths and DALYs using the decomposition methods based on three explanatory components: population aging, population growth, and age-specific rates (epidemiological changes).^14^ All statistical tests were two-sided. The analyses were performed using Statistical Analysis System (version 9.4, SAS Institute Inc., Cary, NC, USA), Joinpoint Regression Program (version 5.0.2, the National Cancer Institute, Rockville, MD, USA), and R (version 4.3.2, R Foundation, Vienna, Austria).

## 3 Results

### 3.1 Burden of enteric infections in China, 2021

**Fig. 1:**
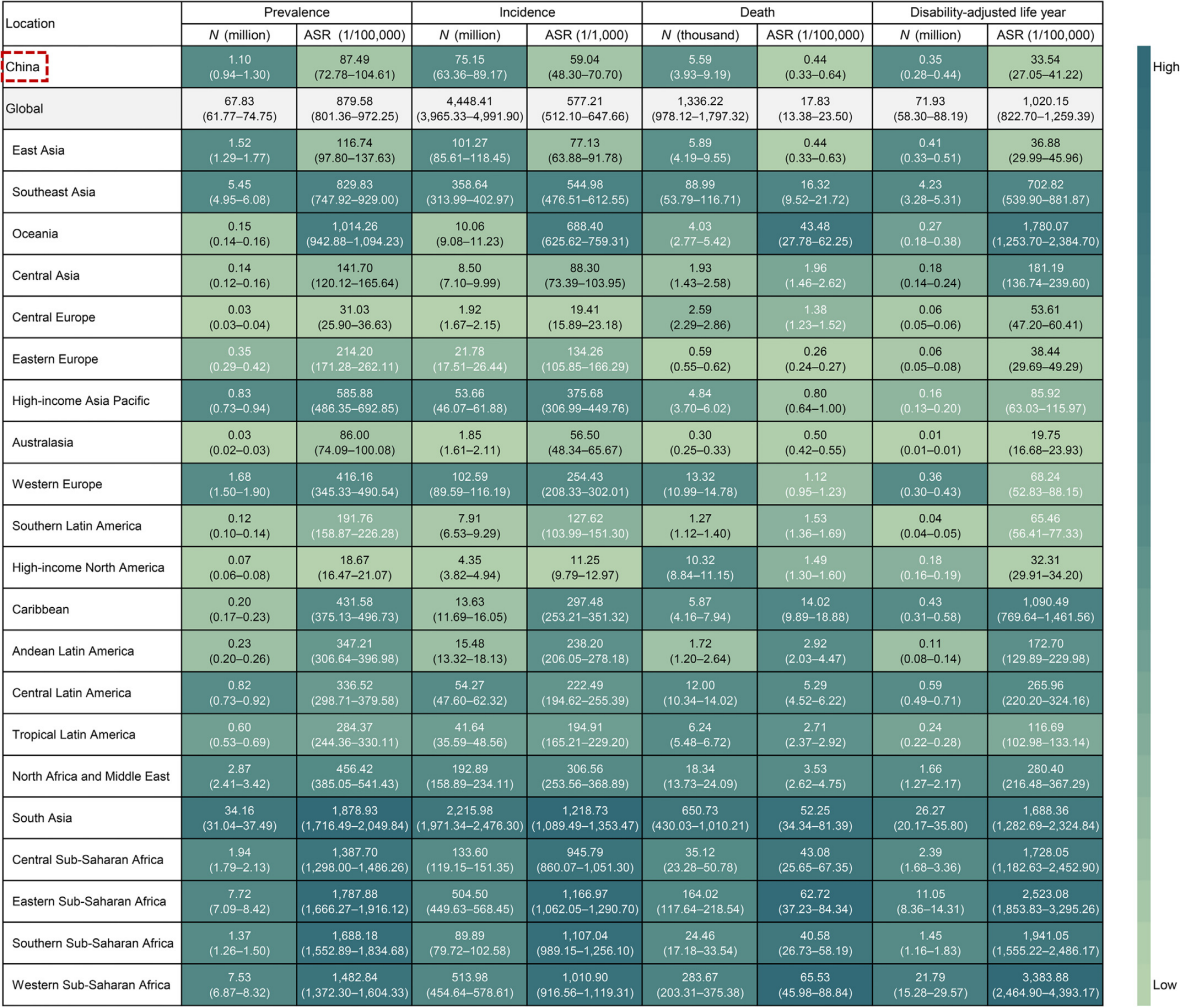
Number, age-standardized prevalence, incidence, death, disability-adjusted life year rates of enteric infections by regions in 2021. *Abbreviation*: ASR, age-standardized rate.

In 2021, the incidence cases of enteric infections reached 75.15 million, and 5,590 deaths were identified in China. The corresponding age-standardized incidence and mortality rates were 59.04 per 100,000 and 0.44 per 100,000, which were one-tenth and one-fortieth of the global average. Age-standardized incidence and mortality rates in China were both ranked relatively low among the 21 GBD regions, with ASRs slightly higher than Australasia (ASIR: 56.50 per 100,000, ranked 19/21), and eastern Europe (ASMR: 0.26 per 100,000, ranked 21/21), respectively. Simultaneously, there were 1.10 million prevalence cases affected by enteric infections resulted in 0.35 million DALYs. Age-standardized prevalence and DALY rates presented significant regional disparities, with only Australasia (ASPR: 86.00 per 100,000; ASDR: 19.75 per 100,000) and high-income North America (ASPR: 18.67 per 100,000; ASDR: 32.31 per 100,000) having lower estimates than China (ASPR: 87.49 per 100,000; ASDR: 33.54 per 100,000) (Fig. 1).

### 3.2 Trends in burden of enteric infections in China, 1990 to 2021

Significant decreases in prevalence cases (−76.87%), incidence cases (−74.12%), deaths (−94.09%), and DALYs (−95.60%) were observed in China from 1990 to 2021. Simultaneously, there were significant decreasing trends in corresponding ASPR (AAPC: −4.91%), ASIR (AAPC: −4.59%), ASMR (AAPC: −9.64%) and ASDR (AAPC: −9.46%). In countries with advanced health system, decreases in prevalence cases (−7.02%) and DALYs (−35.29%) were observed with an increase in deaths (90.41%) for enteric infections. Largest decreases across four health system level in prevalent cases (−43.31%), incident cases (−37.90%), deaths (−77.60%) and DALYs (−84.33%) were observed in countries with basic health system. In countries with limited and minimal health system, decreases in deaths and DALYs were observed with an increase in prevalent and incident cases for enteric infections. Generally decreasing temporal trends in ASPR, ASIR, ASMR and ASDR were observed across four health system level. Basic health system presented larger AAPCs in ASPR (−2.33%), ASIR (−2.06%), ASMR (−6.05%) and ASDR (−6.05%), while the minimal AAPC of ASPR showed in limited health system (−0.52%), the minimal AAPC of ASIR in minimal health system (−0.27%), the minimal AAPC of ASMR (−1.12%) and ASDR (−2.86%) in advanced health system. China presented larger reductions in prevalence cases, incidence cases, deaths and DALYs as well as AAPCs for ASPR, ASIR, ASMR, and ASDR than all four health system levels (Table 1). There were no noticeable differences between both measures between male and female (Table 2, 3).

**Table 1:** Change in number, age-standardized prevalence, incidence, death, disability-adjusted life year rates of enteric infections between 1990 and 2021 in China and four health system leveling.

Measure	*N* (95% UI)		Percent change (95% UI)	ASR, per 100,000 (95% UI)		AAPC (95% CI)
	1990	2021	1990–2021	1990	2021	1990–2021
Prevalence (million)						
China	4.77 (4.04–5.67)	1.10 (0.94–1.30)	−76.87 (−79.52, −73.77)	418.11 (353.01–496.68)	87.49 (72.78–104.61)	−4.91 (−5.02, −4.80)
Advanced health system	4.02 (3.39–4.75)	3.74 (3.28–4.26)	−7.02 (−13.04, −1.18)	350.89 (290.30–419.03)	272.88 (228.12–322.77)	−0.80 (−0.86, −0.74)
Basic health system	18.57 (16.11–21.29)	10.52 (9.33–11.87)	−43.31 (−46.22, −39.66)	763.13 (670.49–869.43)	366.81 (321.09–419.71)	−2.33 (−2.41, −2.25)
Limited health system	35.50 (31.55–39.69)	48.64 (44.35–53.32)	37.02 (28.83, 46.74)	2,011.67 (1,824.78–2,210.49)	1,713.31 (1,573.23–1,860.88)	−0.52 (−0.56, −0.48)
Minimal health system	2.94 (2.70–3.19)	4.89 (4.52–5.32)	66.34 (57.01, 75.59)	1,690.64 (1,568.57–1,815.15)	1,422.93 (1,335.73–1,518.88)	−0.58 (−0.65, −0.52)
Incidence (million)						
China	290.38 (239.86–344.17)	75.15 (63.36–89.17)	−74.12 (−77.02, −70.53)	254.82 (211.15–301.44)	59.04 (48.30–70.70)	−4.59 (−4.72, −4.47)
Advanced health system	254.26 (210.32–300.77)	236.39 (203.65–271.46)	−7.03 (−13.11, 0.05)	221.76 (181.24–265.77)	173.14 (143.14–205.16)	−0.78 (−0.85, −0.72)
Basic health system	1,125.46 (957.07–1,295.50)	698.91 (605.84–806.10)	−37.90 (−41.47, −33.46)	463.07 (396.78–530.63)	242.70 (207.47–282.10)	−2.06 (−2.18, −1.95)
Limited health system	2,198.33 (1,907.77–2,473.21)	3,179.04 (2,839.81–3,559.14)	44.61 (34.16, 57.47)	1,250.98 (1,110.41–1,397.78)	1,118.68 (1,007.28–1,238.26)	−0.36 (−0.39, −0.33)
Minimal health system	178.71 (159.53–201.02)	331.91 (295.34–372.76)	85.73 (74.14, 98.24)	1,036.46 (938.53–1,150.03)	959.13 (875.22–1,057.41)	−0.27 (−0.31, −0.23)
Death (thousand)						
China	94.66 (70.43–117.00)	5.59 (3.93–9.19)	−94.09 (−95.83, −91.22)	9.64 (6.98–12.12)	0.44 (0.33–0.64)	−9.64 (−9.99, −9.29)
Advanced health system	17.84 (15.44–21.21)	33.97 (28.45–37.30)	90.41 (57.17, 121.64)	1.60 (1.37–1.97)	1.13 (0.97–1.23)	−1.12 (−1.45, −0.78)
Basic health system	573.63 (453.33–713.90)	128.48 (86.87–163.19)	−77.60 (−83.07, −72.21)	31.16 (22.71–41.63)	4.52 (3.19–5.61)	−6.05 (−6.19, −5.92)
Limited health system	2,365.55 (1,887.73–2,999.43)	1,001.55 (725.48–1,402.43)	−57.66 (−65.80, −47.74)	217.89 (162.27–302.26)	52.19 (34.87–76.63)	−4.56 (−4.72, −4.40)
Minimal health system	256.03 (184.76–314.44)	171.50 (119.27–230.65)	−33.02 (−49.45, −15.07)	190.55 (130.23–253.05)	71.00 (45.16–98.40)	−3.13 (−3.21, −3.05)
Disability-adjusted life year (million)						
China	7.94 (6.14–9.77)	0.35 (0.28–0.44)	−95.60 (−96.69, −94.19)	726.41 (559.34–893.93)	33.54 (27.05–41.22)	−9.46 (−9.82, −9.10)
Advanced health system	1.53 (1.28–1.86)	0.99 (0.85–1.16)	−35.29 (−45.95, −25.72)	148.39 (124.42–183.11)	60.67 (50.39–73.82)	−2.86 (−3.01, −2.72)
Basic health system	41.88 (35.04–48.73)	6.56 (5.35–7.92)	−84.33 (−86.93, −81.21)	1,763.94 (1,457.36–2,094.99)	256.38 (209.82–309.94)	−6.05 (−6.26, −5.85)
Limited health system	151.37 (123.16–177.41)	51.91 (42.10–63.49)	−65.70 (−71.52, −59.01)	8,433.90 (6,809.64–1,0546.74)	1,985.09 (1,594.11–2,500.85)	−4.58 (−4.68, −4.49)
Minimal health system	19.55 (14.07–24.01)	12.42 (8.98–16.89)	−36.48 (−52.66, −17.98)	9,309.65 (6,761.08–1,1541.18)	3,071.67 (2,188.19–4,070.65)	−3.52 (−3.66, −3.38)

*Abbreviations*: ASR, age-standardized rate; UI, uncertainty interval; AAPC, average annual percent change; CI, confidence interval.

**Table 2:** Change in number, age-standardized prevalence, incidence, death, disability-adjusted life year rates of enteric infections in males between 1990 and 2021 in China and four health system leveling.

Measure	*N* (95% UI)		Percent change (95% UI)	ASR, per 100,000 (95% UI)		AAPC (95% CI)
	1990	2021	1990–2021	1990	2021	1990–2021
Prevalence (million)						
China	2.53 (2.15–2.99)	0.62 (0.52–0.73)	−75.49 (−78.21, −72.48)	424.36 (359.03–502.01)	96.60 (79.95–116.04)	−4.65 (−4.77, −4.53)
Advanced health system	1.94 (1.62–2.30)	1.83 (1.59–2.10)	−5.79 (−11.64, −0.08)	342.19 (282.59–409.90)	275.85 (230.39–325.44)	−0.68 (−0.75, −0.62)
Basic health system	9.51 (8.23–10.90)	5.31 (4.70–6.01)	−44.14 (−47.22, −40.32)	761.67 (667.39–867.54)	364.35 (318.50–417.42)	−2.35 (−2.42, −2.28)
Limited health system	17.30 (15.20–19.55)	23.59 (21.38–26.08)	36.34 (27.18, 46.69)	1,887.10 (1,708.50–2,098.70)	1,635.65 (1,494.64–1,784.56)	−0.46 (−0.50, −0.42)
Minimal health system	1.52 (1.39–1.65)	2.53 (2.33–2.75)	66.11 (56.15, 76.08)	1,759.67 (1,633.92–1,890.21)	1,491.56 (1,399.54–1,597.36)	−0.54 (−0.62, −0.47)
Incidence (million)						
China	153.17 (126.37–182.68)	42.17 (35.19–49.97)	−72.47 (−75.54, −68.98)	257.78 (212.67–306.78)	65.31 (53.34–78.45)	−4.32 (−4.42, −4.21)
Advanced health system	122.00 (100.60–144.66)	115.37 (98.37–133.86)	−5.43 (−11.59, 1.75)	215.56 (175.46–257.90)	174.90 (144.74–206.64)	−0.66 (−0.73, −0.59)
Basic health system	573.48 (487.19–662.56)	352.86 (303.76–406.53)	−38.47 (−42.35, −34.13)	459.74 (393.43–528.01)	241.42 (206.24–279.17)	−2.05 (−2.16, −1.94)
Limited health system	1,066.42 (917.09–1,206.74)	1,540.54 (1,368.09–1,736.75)	44.46 (33.35, 58.39)	1,167.68 (1,027.48–1,310.95)	1,069.57 (957.86–1,192.21)	−0.28 (−0.32, −0.24)
Minimal health system	92.25 (82.40–103.41)	170.54 (150.97–193.14)	84.87 (72.68, 97.67)	1,075.28 (968.02–1,198.51)	998.20 (905.56–1,108.08)	−0.25 (−0.29, −0.20)
Death (thousand)						
China	49.45 (33.18–67.42)	2.96 (1.90–4.81)	−94.01 (−96.13, −89.92)	9.90 (6.45–13.60)	0.48 (0.33–0.72)	−9.30 (−9.69, −8.91)
Advanced health system	8.86 (7.38–11.06)	13.95 (12.47–15.03)	57.51 (26.14, 88.57)	1.76 (1.46–2.22)	1.20 (1.08–1.30)	−1.20 (−1.48, −0.91)
Basic health system	291.88 (221.11–378.04)	62.58 (40.52–84.89)	−78.56 (−83.49, −72.24)	31.98 (21.86–43.59)	4.66 (3.16–6.16)	−6.03 (−6.18, −5.89)
Limited health system	1,196.42 (884.88–1,579.49)	483.74 (326.07–729.44)	−59.57 (−67.95, −49.45)	210.02 (135.27–313.84)	50.54 (31.65–81.02)	−4.53 (−4.76, −4.30)
Minimal health system	146.13 (101.67–186.85)	93.01 (61.75–132.31)	−36.35 (−54.35, −14.83)	205.57 (129.23–278.34)	80.69 (45.23–121.28)	−2.97 (−3.05, −2.89)
Disability-adjusted life year (million)						
China	4.17 (2.83–5.61)	0.19 (0.15–0.25)	−95.37 (−96.69, −93.18)	722.89 (489.90–969.05)	35.26 (27.38–44.98)	−9.35 (−9.72, −8.99)
Advanced health system	0.80 (0.64–1.00)	0.47 (0.40–0.56)	−41.82 (−52.22, −31.78)	157.20 (126.11–198.07)	62.50 (51.71–76.45)	−2.93 (−3.09, −2.78)
Basic health system	21.89 (17.12–27.32)	3.45 (2.74–4.39)	−84.26 (−87.28, −80.19)	1,792.73 (1,395.25–2,246.69)	265.31 (213.97–330.26)	−6.01 (−6.09, −5.93)
Limited health system	78.28 (61.64–96.08)	27.08 (20.78–35.42)	−65.41 (−72.33, −57.28)	8,392.08 (6,360.67–10,976.17)	2,023.97 (1,525.72–2,765.50)	−4.50 (−4.66, −4.34)
Minimal health system	11.33 (7.82–14.42)	6.70 (4.55–9.49)	−40.86 (−57.23, −20.42)	10,505.62 (7,489.42–13,332.28)	3,392.38 (2,261.05–4,824.69)	−3.59 (−3.73, −3.45)

*Abbreviations*: ASR, age-standardized rate; UI, uncertainty interval; AAPC, average annual percent change; CI, confidence interval.

**Table 3:** Change in number, age-standardized prevalence, incidence, death, disability-adjusted life year rates of enteric infections in females between 1990 and 2021 in China and four health system leveling.

Measure	*N* (95% UI)		Percent change (95% UI)	ASR, per 100,000 (95% UI)		AAPC (95% CI)
	1990	2021	1990–2021	1990	2021	1990–2021
Prevalence (million)						
China	2.25 (1.90–2.68)	0.48 (0.41–0.57)	−78.42 (−81.02, −75.27)	410.96 (345.48–490.68)	77.37 (64.75–92.09)	−5.24 (−5.35, −5.12)
Advanced health system	2.08 (1.76–2.45)	1.91 (1.69–2.16)	−8.16 (−14.23, −2.12)	359.18 (298.25–428.81)	270.09 (226.26–318.61)	−0.91 (−0.97, −0.85)
Basic health system	9.06 (7.88–10.39)	5.21 (4.63–5.88)	−42.44 (−45.54, −38.59)	763.47 (669.79–871.13)	368.36 (322.72–421.93)	−2.32 (−2.43, −2.20)
Limited health system	18.20 (16.27–20.13)	25.05 (22.93–27.30)	37.67 (29.47, 47.38)	2,141.54 (1,942.45–2,346.49)	1,787.58 (1,649.24–1,934.14)	−0.59 (−0.62, −0.57)
Minimal health system	1.42 (1.29–1.54)	2.36 (2.18–2.58)	66.60 (56.63, 77.65)	1,625.01 (1,506.04–1,744.95)	1,356.31 (1,272.56–1,450.40)	−0.59 (−0.64, −0.54)
Incidence (million)						
China	137.21 (112.85–162.24)	32.97 (27.86–38.97)	−75.97 (−78.92, −72.31)	251.34 (208.26–296.90)	52.11 (43.13–61.96)	−4.93 (−5.06, −4.80)
Advanced health system	132.26 (109.98–156.38)	121.03 (104.41–138.49)	−8.50 (−14.72, −1.40)	227.67 (186.01–273.40)	171.50 (141.65–203.35)	−0.90 (−0.97, −0.83)
Basic health system	551.99 (470.25–633.15)	346.05 (299.72–396.49)	−37.31 (−40.96, −32.72)	465.80 (400.52–531.86)	243.39 (207.86–282.85)	−2.07 (−2.20, −1.94)
Limited health system	1,131.91 (991.15–1,270.51)	1,638.50 (1,468.86–1,822.38)	44.75 (34.39, 56.95)	1,337.93 (1,196.55–1,498.22)	1,166.26 (1,053.16–1,282.90)	−0.45 (−0.48, −0.41)
Minimal health system	86.46 (76.88–97.23)	161.37 (144.38–180.53)	86.64 (73.67, 100.41)	999.59 (897.77–1,112.62)	921.11 (839.94–1,018.35)	−0.26 (−0.30, −0.23)
Death (thousand)						
China	45.20 (34.62–56.58)	2.63 (1.62–5.01)	−94.18 (−96.25, −90.41)	9.52 (7.09–12.18)	0.41 (0.29–0.66)	−9.67 (−10.01, −9.33)
Advanced health system	8.98 (7.58–11.45)	20.02 (16.10–22.52)	122.84 (75.33, 167.68)	1.46 (1.18–1.99)	1.07 (0.91–1.19)	−1.04 (−1.42, −0.66)
Basic health system	281.75 (212.44–386.63)	65.90 (38.91–95.70)	−76.61 (−84.04, −69.16)	30.51 (20.72–44.99)	4.38 (2.83–6.08)	−6.07 (−6.20, −5.93)
Limited health system	1,169.12 (858.45–1,621.00)	517.81 (314.78–852.27)	−55.71 (−68.64, −40.64)	225.28 (145.06–337.28)	53.26 (29.39–92.06)	−4.55 (−4.71, −4.39)
Minimal health system	109.90 (79.25–139.71)	78.49 (52.89–107.23)	−28.58 (−48.80, −5.14)	175.27 (104.90–269.24)	62.59 (33.18–96.91)	−3.25 (−3.37, −3.13)
Disability-adjusted life year (million)						
China	3.77 (3.03–4.63)	0.16 (0.12–0.21)	−95.86 (−97.01, −94.40)	732.20 (586.66–898.12)	31.87 (25.30–40.50)	−9.62 (−9.95, −9.30)
Advanced health system	0.72 (0.59–0.95)	0.52 (0.45–0.61)	−28.01 (−43.89, −14.18)	139.46 (112.76–187.25)	59.11 (48.97–72.79)	−2.72 (−2.95, −2.49)
Basic health system	19.99 (16.76–23.80)	3.12 (2.42–4.04)	−84.41 (−87.57, −80.95)	1,735.40 (1,418.68–2,116.95)	246.82 (200.25–301.97)	−6.11 (−6.27, −5.95)
Limited health system	73.08 (57.59–89.81)	24.84 (18.60–32.62)	−66.01 (−73.72, −57.68)	8,467.58 (6,354.65–11,528.95)	1,935.61 (1,414.75–2,679.66)	−4.67 (−4.79, −4.54)
Minimal health system	8.23 (6.16–10.14)	5.72 (4.08–7.71)	−30.44 (−49.24, −6.89)	8,095.13 (5,837.40–10,496.26)	2,770.07 (1,884.02–3,723.91)	−3.40 (−3.57, −3.24)

*Abbreviations*: ASR, age-standardized rate; UI, uncertainty interval; AAPC, average annual percent change; CI, confidence interval.

### 3.3 Trends in burden of enteric infections by etiology in China, 1990 to 2021

For specific etiologies, the ASIR of diarrheal diseases showed a substantial decrease from 254.58 in 1990 to 58.94 in 2021, with an AAPC of −4.60%. The decline in typhoid and paratyphoid fevers was also notable. For typhoid fever, the ASPR and ASIR decreased both by 2.94%, respectively. Paratyphoid fever saw a decrease of 2.63% in ASPR and 2.64% in ASIR. For diarrheal diseases, the ASMR decreased from 9.32 to 0.34 between 1990 and 2021, with an AAPC of −10.17%. The ASDR dropped from 703.47 to 26.68, with an AAPC of −10.06%. The ASMR of typhoid and paratyphoid fevers also presented a decreasing trend, with AAPCs of −3.78% for typhoid fever, and −3.73% for paratyphoid fever, respectively. There was no noticeable difference in these trends between males and females (Table 4).

**Table 4:** Trends in age-standardized prevalence, incidence, mortality and disability-adjusted life year rates of enteric infections by etiologies in China, 1990 to 2021.

Etiologies	ASPR		AAPC	ASIR		AAPC	ASMR		AAPC	ASDR		AAPC
	1990	2021	1990–2021	1990	2021	1990–2021	1990	2021	1990–2021	1990	2021	1990–2021
Total												
Diarrheal diseases	416.83	86.96	−4.92	254.58	58.94	−4.60	9.32	0.34	−10.17	703.47	26.68	−10.06
Typhoid and paratyphoid	1.29	0.53	−2.84	0.23	0.09	−2.82	0.21	0.07	−3.78	16.45	5.02	−3.89
Typhoid fever	0.90	0.36	−2.94	0.14	0.06	−2.94	0.16	0.05	−3.83	11.85	3.56	−3.96
Paratyphoid fever	0.39	0.17	−2.63	0.09	0.04	−2.64	0.06	0.02	−3.70	4.60	1.47	−3.64
Invasive Non−typhoidal Salmonella (iNTS)	—	—	—	—	—	—	0.03	0.01	−6.02	2.50	0.24	−7.25
Other intestinal infectious diseases	0.01	0.01	−0.10	0.00	0.00	−0.10	0.07	0.03	−2.61	3.98	1.60	−2.92
Male												
Diarrheal diseases	422.98	96.04	−4.66	257.53	65.2	−4.32	9.56	0.37	−9.93	698.43	27.96	−9.84
Typhoid and paratyphoid	1.38	0.56	−2.87	0.25	0.1	−2.85	0.24	0.07	−3.86	18.34	5.45	−3.86
Typhoid fever	0.97	0.38	−2.94	0.15	0.06	−2.95	0.17	0.05	−3.80	13.12	3.86	−3.95
Paratyphoid fever	0.41	0.18	−2.68	0.09	0.04	−2.69	0.06	0.02	−3.73	5.22	1.59	−3.81
Invasive Non−typhoidal Salmonella (iNTS)	—	—	—	—	—	—	0.03	0.01	−5.33	2.11	0.25	−6.61
Other intestinal infectious diseases	0.01	0.01	−0.11	0.00	0.00	−0.05	0.07	0.03	−2.63	4.01	1.60	−2.94
Female												
Diarrheal diseases	409.78	76.87	−5.25	251.13	52.02	−4.94	9.22	0.31	−10.34	710.93	25.50	−10.16
Typhoid and paratyphoid	1.19	0.49	−2.82	0.21	0.09	−2.79	0.19	0.06	−3.69	14.37	4.54	−3.78
Typhoid fever	0.83	0.33	−2.93	0.13	0.05	−2.93	0.14	0.04	−3.74	10.45	3.21	−3.88
Paratyphoid fever	0.36	0.16	−2.57	0.08	0.04	−2.58	0.05	0.02	−3.52	3.92	1.33	−3.58
Invasive Non−typhoidal Salmonella (iNTS)	—	—	—	—	—	—	0.04	0.00	−6.65	2.95	0.23	−7.86
Other intestinal infectious diseases	0.01	0.01	−0.13	0.00	0.00	−0.13	0.07	0.03	−2.65	3.96	1.60	−3.03

*Abbreviations*: ASPR, age-standardized prevalence rate; ASIR, age-standardized incidence rate; ASMR, age-standardized mortality rate; ASDR, age-standardized disability-adjusted life year rate; AAPC, average annual percent change.

### 3.4 Decomposition analysis for burden of enteric infections, 1990 to 2021

**Fig. 2:**
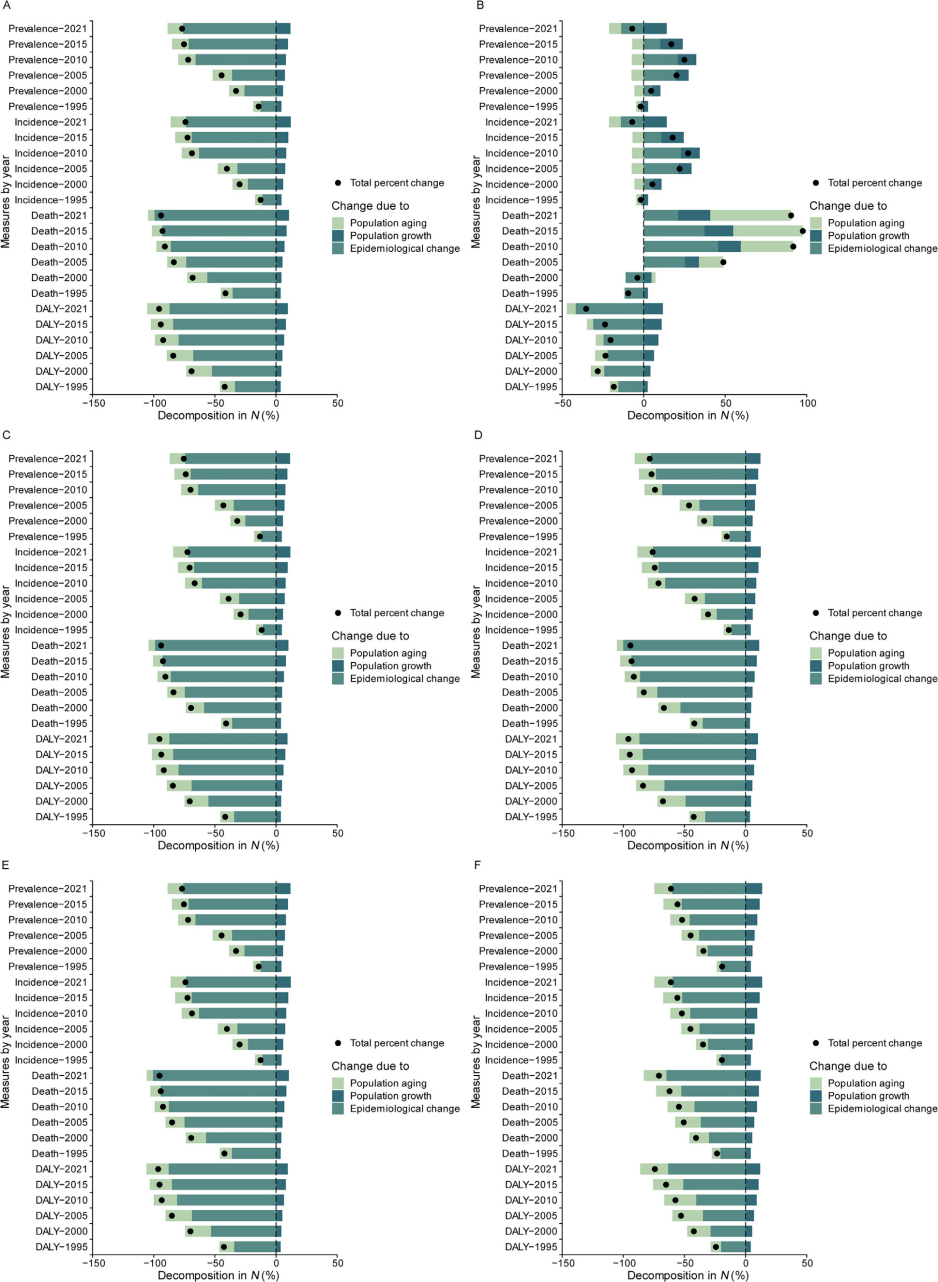
Decomposition analysis of changes in prevalence, incidence, death and DALY of enteric infections in China (A) and Advanced Health System (B), in males (C) and females (D) in China, and for diarrheal diseases (E) and typhoid and paratyphoid fevers (F) in China, compared with 1990. *Abbreviation*: DALY, disability-adjusted life year.

In decomposition analysis, the results showed that total change in incident cases, patients, deaths, DALYs of enteric infections was primarily driven by epidemiological changes (age-specific rates), followed by population aging in China, with an escalating decrease from 1995 to 2021. In contrast, population growth had an opposing impact with a similar escalating rate by 2021. Specifically in 2021, epidemiological change accounted for −75.81% change in prevalence cases, −73.55% change in incidence cases, −99.31% change in deaths, and −86.96% change in DALYs. Population aging contributed to −12.85% change in prevalence cases, −12.60% change in incidence cases, −5.33% change in deaths and −18.34% change in DLAYs. Population growth in turn increased prevalent cases by 11.78%, incidence cases by 12.03%, deaths by 10.55%, and DALYs by 9.70% (Fig. 2A). Within Advanced health systems, the total change in enteric infections was generally driven positively by population growth in prevalence cases, followed by population aging as the major driver in deaths. Conversely, epidemiological change was the main driver of change in DALYs by 2021 (Fig. 2B). Similar changing patterns of the drivers for the overall burden of enteric infections were observed by sex (Fig. 2C, D), in diarrheal diseases (Fig. 2E), and in typhoid and paratyphoid fevers (Fig. 2F).

### 3.5 Burden of enteric infections by sex and age in China, 2021

**Fig. 3:**
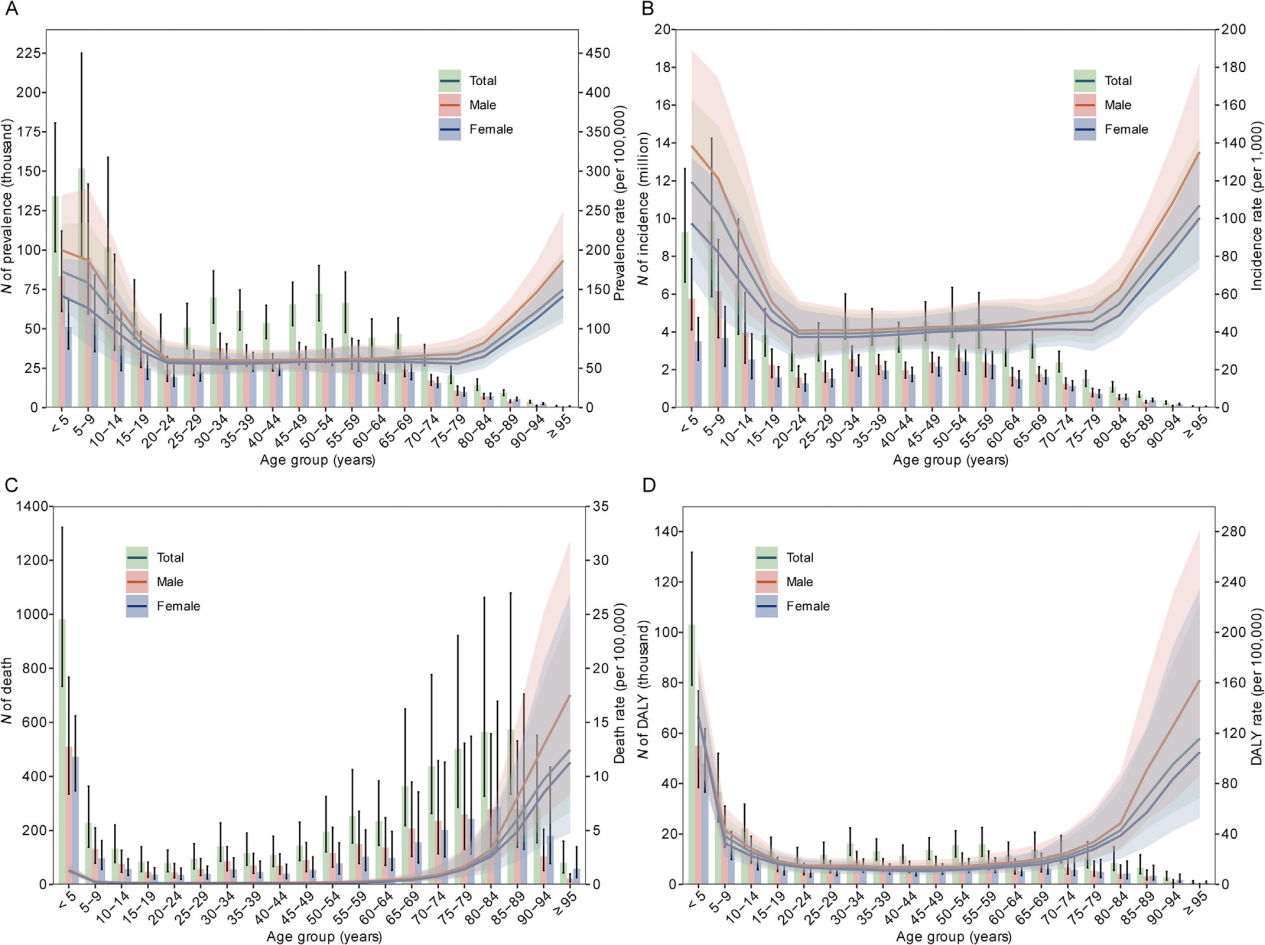
Number and rate of prevalence (A), incidence (B), death (C) and DALY (D) of enteric infections by sex and age in 2021. *Abbreviation*: DALY, disability-adjusted life year.

In 2021, the results show that incidence of enteric infections was the highest in children aged between 5 and 9 years old, with over 9.8 million incident cases for both sexes, 63% of them being males. This was followed by the younger age groups under 5 years old by 5.8 million cases in males and 3.5 million females; the incidence decreased in general incrementally by increasing age. In the overall incidence of enteric infections and across all age groups, the incidence rate in males is higher than that in females. Similar patterns were seen in prevalence, males and younger age groups being more predominant in contributing to enteric infections (Table 2, 3, Fig. 3A, B). On the other hand, the oldest age groups had the highest mortality rates due to enteric infections: 279 deaths were recorded for males and 295 deaths for females in the 85–89 years age group. Mortality rates for the youngest age groups were remarkably low, with under-5 mortality rates standing at 1.2 per 100,000 for males and 1.3 per 100,000 for females. Overall male's mortality was higher across all age groups compared to females. Highest DALYs were observed in children under 5 years of age with a total of 103,015 from both sex (53.4% from males). These DALYs accounted for 41.8% (103,015/246,299) of the total DALYs after 5 years of age, with dramatically decrease to about one-third of that in 5–9 years' age group (36,660). Generally, males had higher DALYs than females across most age groups, except for children aged 5–9 years (Fig. 3C, D).

## 4 Discussion

This study assessed the temporal trends in the burden of enteric infections in China from 1990 to 2021. In 2021, China reported 75.15 million incidence cases and 5,590 deaths from enteric infections with significant decrease in prevalence cases, incidence cases, deaths and DALYs over the past 30 years. Simultaneously, consistent decreasing trends were observed in ASPR, ASIR, ASMR and ASDR of enteric infections in China. Diarrheal diseases were consistently the main contributor of the burden of enteric infections with the highest decrease in ASPR, ASIR, ASMR and ASDR across all etiologies of enteric infections. Decomposition analysis showed that the decrease in prevalence cases, incidence cases deaths, DALYs of enteric infections was primarily driven epidemiological changes (age-specific rates). Difference in enteric infections burden patterns were observed across age groups, with higher incidence rates and DALY rates were identified in child under 15 years and older population aged 85 and above in 2021.

In 2021, the ASIR and ASMR of enteric infections in China were significantly lower than the global averages, being one-tenth and one-fortieth of the global rates. The burden of enteric infections in China ranked relatively low among the 21 GBD regions, with slightly higher ASIR than Australasia and slightly higher ASMR than eastern Europe. These regional differences in the epidemiology of enteric infections may be linked to the disparities in sanitary conditions, medical resources, and preventive measures.^15^, ^16^, ^17^ Interventions to promote water, sanitation, and hygiene, including safe drinking water, use of improved sanitation, and hand washing with soap, can reduce the transmission of enteric infections. Over the past few decades, systematic enhancements of water and sanitation infrastructure in China have contributed significantly to the sustained low burden of enteric infections.^18^^,^^19^ Specifically, access to safe water and sanitary toilets in China increased significantly from 45.7% and 18.7% to 91.3% and 78.5%, respectively, from 2000 to 2020. Simultaneously, a low-level health system can impact the management and reporting of enteric infections. In contrast, China has better access to medical resources and more effective treatment, evidenced by the lower mortality-to-incidence ratio.

From 1990 to 2021, China has made significant strides in reducing the burden of enteric infections, with substantial decreases in prevalence cases, incidence case, deaths, and DALYs. The decreases were mainly driven by epidemiological changes. These declines are mirrored in the corresponding ASPR, ASIR, ASMR, and ASDR. The decreasing trend in the burden of enteric infections in China is in line with the global trend,^2^^,^^3^ but the pattern of decrease is noteworthy when considered health system level. The burden of enteric infections in China was similar to countries and territories with advanced health system in 1990, but significantly lower in 2021. In countries and territories with advanced health systems, decreases in prevalence cases and DALYs, but an increase in deaths for enteric infections was determined. The increase in deaths was mainly driven by population aging and epidemiological change, then population growth. Aging population aged ≥ 65 years is more susceptible to certain enteric (including foodborne) infections such as *Campylobacter* and *Salmonella* bacteremia, which may contribute to this increase in deaths.^20^ The largest decreases in prevalence cases, incidence cases, deaths, and DALYs were observed in countries with basic health systems, convincing that improvement of foundational health infrastructures can be highly effective in reducing enteric infection burdens. In contrast, countries with limited and minimal health systems showed decreases in deaths and DALYs but increases in prevalence and incidence cases, highlighting the importance of robust health systems in controlling the spread of enteric infections. The consistent decreasing trends in ASPR, ASIR, ASMR, and ASDR across different health system levels underscore the global impact of health system strength on enteric infection outcomes.^21^, ^22^, ^23^ Larger percent changes and AAPCs in China compared to all four health system levels highlight the efficiency and success in related health strategies.

China has adopted a multifaceted approach to prevent enteric infections, encompassing national notifiable infectious disease surveillance, vaccination, personal and environmental hygiene, training of medical and public health professionals.^24^, ^25^, ^26^, ^27^ The importance of handwashing and other personal hygiene practices was emphasized at the national level to reduce the spread of enteric infections.^28^ In healthcare settings, strict infection control measures are implemented, including contact precautions for patients with illnesses transmitted by direct contact or through articles in the patient's environment.^29^ Surveillance systems are in place to monitor outbreaks and manage cases effectively, which is crucial for early detection and response.^25^^,^^29^ Moreover, non-pharmaceutical interventions during the COVID-19, including hygiene campaigns, movement restrictions, may have reduced enteric transmission in 2020 and 2021.^30^ These measures are part of a broader strategy to enhance the health system in China, reflecting a comprehensive and systematic effort against enteric infections. The findings in this study underscore the importance of investing in health systems and the potential for significant reductions in enteric infection burdens when effective measures are implemented.

Regarding the specific etiology of enteric infections in China, diarrheal diseases were consistently the leading contributor with the highest decrease in ASPR, ASIR, ASMR, and ASDR. The ASIR of diarrheal diseases decreased from 254.58 in 1990 to 58.94 in 2021. Typhoid and paratyphoid fevers, as the severe type of enteric infections, are classified as Class B of notifiable infectious diseases in China.^8^^,^^24^, ^25^, ^26^, ^27^ Our study observed that the ASIR and ASMR decreased by 2.94% and 3.83% for typhoid fever, respectively, and 2.64% and 3.70% for paratyphoid fever, respectively. Similar to the overall pattern of enteric infections, epidemiological changes have played a crucial role in the decrease of cases of diarrheal diseases and typhoid and paratyphoid fever. Moreover, the burden of iNTS and other intestinal infectious diseases followed a similar decreasing trend, with ASDR decreasing by 7.25% and 2.92%, respectively. Antimicrobial resistance in enteric pathogens (e.g., *Salmonella, Shigella*) poses challenges to the control of enteric infections and may exacerbate mortality and DALYs. This study cannot directly assess antimicrobial resistance, but rising resistance in China may offset gains in infection control.^31^^,^^32^ These findings highlight that sustained efforts are needed for targeted prevention and control to reduce the incidence of diarrheal diseases and mortality from typhoid and paratyphoid fevers.

In 2021, the highest incidence rate and DALY rate of enteric infections in China were observed in children under 5, who are more susceptible due to immature immune systems and malnutrition, consistent with previous studies. As children age, the incidence decreases, possibly due to the development of immunity through natural exposure to pathogens.^2^^,^^8^^,^^10^ The DALYs is highest in children under 5, with a total of 103,014.9 in both sexes, 53% of them being males, accounting for approximately 30% of total DALYs. This burden dramatically drops after the age of 5 and continues to decrease throughout adolescence before flattening out in adulthood with a slight raise at middle age. Progressively after the age of 70, the incidence rate, mortality rate and DALY rate increased dramatically. The high mortality from enteric infections in the older age groups, particularly those aged 85 and above, is attributed to the increased susceptibility of the older adults to infectious diseases due to a weakened immune system and the presence of comorbidities. The older adults are also more likely to have reduced access to proper healthcare, which can delay diagnosis and treatment, leading to higher mortality rates. Additionally, the increasing antibiotic resistance in enteric pathogens can result in less effective treatments, especially in older adults who may not tolerate more aggressive therapies well.^2^^,^^9^^,^^15^^,^^19^^,^^20^ The patterns suggest that in China, enteric infections cause the highest burden among young children, with high incidence, prevalence, and mortality in this age group, while geriatric groups, particularly those aged 85+ years, also present a high mortality from these infections. Generally, males tend to bear a higher burden in comparison with females on most measures analyzed. The higher incidence in males across all age groups might be linked to behavioral differences and a higher likelihood of engaging in activities that increase exposure to contaminated environments.^6^^,^^7^^,^^17^^,^^33^ Enhanced interventions and health promotion strategies should be targeted at both young and older populations, and among males, such as expanding rotavirus vaccination in children < 5 years and geriatric antibiotic stewardship programs to reduce resistance-related mortality.

This study is subject to several limitations. First, the data sourced from the GBD 2021, which may have inherent biases or gaps in reporting, particularly for certain regions or pathogens. Second, the estimates of incidence, mortality and DALY were subject to the variability and potential inaccuracies in the underlying data sources. Third, the ASRs reported in this study may not fully capture the nuances of changing population structures over time within China. Fourth, the temporal trends were based on aggregated data, which limits the ability to draw specific conclusions about risk factors and the effectiveness of targeted interventions at the provincial level in China. Fifth, this analysis could not differentiate urban-rural disparities due to GBD data constraints. Despite these limitations, the study offers valuable insights into the burden and trends of enteric infections in China and underscores the need for continued efforts and improved control strategies.

## 5 Conclusion

This study provides a comprehensive overview of the burden of enteric infections in China from 1990 to 2021. Decreases in prevalence cases, incidence cases, deaths and DALYs were observed and mainly driven by epidemiological changes. Consistently decreasing trends in ASPR, ASIR, ASMR, and ASDR were determined for enteric infections, diarrheal diseases, and typhoid and paratyphoid fevers. Diarrheal diseases were consistently the main contributor to the burden of enteric infections in China between 1990 and 2021. Higher incidence rates and DALY rates were identified in in children under 15 years and the older population aged 85 and above in 2021. These findings underscore the need for continued efforts for the improvement of the health system, including antibiotic management, and enhanced interventions and health promotion strategies, including scaling pediatric rotavirus vaccination and geriatric-focused interventions.
